# Medical Degrees and Diplomas

**Published:** 1916-11-18

**Authors:** 


### The Hospital

The Workers' Newspaper of Administrative Medicine and Institutional Life, Administration, National Insurance and Health.

No. 1589, Vol. LXI. SATURDAY, NOVEMBER 18, 1916. PRICE ONE PENNY

### MEDICAL DEGREES AND DIPLOMAS.

The public, and small blame to it, is woefully perplexed by the numerous alphabetical decorations with which medical practitioners indicate their titular qualifications. Doctor This and Mister That are not altogether plain sailing, but when it comes to fellowships and memberships and licentiateships the puzzle to the man in the street is beyond solution. Of recent years the question has become still more confused by the setting up of special diplomas in public health, tropical diseases, midwifery, and ophthalmology; and there is, further, the complication of the medical and other societies and the special titles which union with these assures. A Fellowship of the Royal Society of Medicine may well seem as virtuous as a Fellowship of one of the Royal Colleges, and the simple-minded may be puzzled to define the difference between a Member of the British Medical Association and a Member of the Royal College of Surgeons. Even the mere *number* of " letters " attached to a man's name has been regarded as the measure of his greatness, a test which would leave the two-lettered M.D. hopelessly behind the sixteen-lettered licentiate of the conjoined Scottish Colleges. No one may hope fully to inform the lay mind on all these mysteries, nor indeed does it greatly matter. For the layman has usually learnt from practical experience that what is of importance is the man and not his parchments or his titles. These interesting documents are, perhaps legitimately, sources of pride to their possessor and to his female relatives, but they do not go far in the judgment of a censorious world unless supported by practical proofs of capacity. Indeed, the remark has been heard that when presented with some measure of ostentation diplomas and titles are apt to engender the suspicion that the holder has probably little else of which to be proud. And, in truth, rival displays of this order have a juvenile quality at which the rough test of experience is apt to smile or even to grow cynical. At best the dispute is one of medical domestic politics, and if brought to the judgment seat may well provoke from the modern Gallio the reply: " If it be a question of words and names and of your law, look ye to it; for I will be no judge of such matters."

There is, however, one relation of practical life in which medical titles—their worth, value, and significance—force themselves on the attention of laymen, or at least on those laymen who are responsible for the administration of hospitals. Appointments to the medical and surgical staff of a hospital and the conditions which govern these appointments rest in the last resort on the sanction of the lay governors. It is true that in many, if not in most, instances, the laymen accept the direction of the medical committee, but as the final vote is in their hands they cannot escape responsibility; and in any event it is well for them to know exactly what they are doing. Hence, here there must be some lay interest in medical titles and diplomas. It may be said generally that a broad distinction between a degree and a diploma is that the former is conferred by a University and the latter by an examining board. In each case, of course, possession indicates a successful passage of examinations. But the University degree means also a definite course of training, the nature of which is determined by the academic authorities. It is true that preliminary to the diploma also there is a compulsory course of study, but this course is not supervised and controlled by the diploma-granting body nor are the individual teachers under its direction. From this it follows that in a University the training is regarded as valuable in itself, and at least as important as the examinations to which it conducts; whereas an educational plan which aims at a diploma inevitably has constantly to keep its eye on the examination and on the examiners. In the one case the teaching commands the examination while in the other the examination rules the teaching. Again, in every true University there are faculties in Divinity, Arts, Law, and Science, as well as in Medicine. Hence the undergraduate has his being in an atmosphere charged with many interests and calculated therefore to broaden his outlook and to multiply his sympathies. The mere medical school, on the other hand, cannot boast these advantages, and in the very nature of things partakes rather of the character of a technical college. It cannot, therefore, in its atmosphere claim the multiple educational influences which are present in a University. Many may be disposed to say that if the student possesses the knowledge which enables him to pass the qualifying examinations it is of no importance where and how he gets this knowledge. But such view is by no means that of the leading authorities on education. These insist rather on the importance of the training, and place examination in a relatively subordinate position. Such an opinion, too, is a growing quantity, and the influence of it is seen in the attempts to make the University of London a true teaching University instead of a mere examining board. In these circumstances it seems somewhat peculiar that in the majority of hospitals there is a rule that members of the staff, whatever University degrees they possess, shall hold a diploma from one of the examining bodies, a College of Physicians in the one case and a College of Surgeons in the other. The possession of such a diploma does no more than signify that at a certain date the holder reached a certain examination standard. He may have got there only by the skin of his teeth, or, on the other hand, he may have obtained a much higher level than the mere pass. Again, his success may have been due to a course of grinding and cramming fitted to equip him for the day of trial, or it may have followed a real training devoted to the acquisition of real knowledge. But on all these points the diploma is silent. Why, then, is its possession demanded by hospital authorities? The complete answer would be a long story. Tradition enters into it, as does also monopoly and the love of power. The one valid defence for it is that holders of these diplomas come under certain conditions fixed by the bodies which grant them. Whether good or bad it is desirable that the same conditions shall apply to all members of the same staff, and this the rule here discussed ensures. Whether there is not a more sensible method of obtaining this end is another question, and one which will probably be discussed in the not remote future.

